# Effects of Age and Noise Exposure on Proxy Measures of Cochlear Synaptopathy

**DOI:** 10.1177/2331216519877301

**Published:** 2019-09-27

**Authors:** Garreth Prendergast, Samuel Couth, Rebecca E. Millman, Hannah Guest, Karolina Kluk, Kevin J. Munro, Christopher J. Plack

**Affiliations:** 1 Manchester Centre for Audiology and Deafness, The University of Manchester, Manchester Academic Health Science Centre, UK; 2 NIHR Manchester Biomedical Research Centre, Central Manchester University Hospitals NHS Foundation Trust, Manchester Academic Health Science Centre, Manchester, UK; 3 Department of Psychology, Lancaster University, UK

**Keywords:** cochlear synaptopathy, hidden hearing loss, noise-induced hearing loss, speech in noise, psychophysics

## Abstract

Although there is strong histological evidence for age-related synaptopathy in humans, evidence for the existence of noise-induced cochlear synaptopathy in humans is inconclusive. Here, we sought to evaluate the relative contributions of age and noise exposure to cochlear synaptopathy using a series of electrophysiological and behavioral measures. We extended an existing cohort by including 33 adults in the age range 37 to 60, resulting in a total of 156 participants, with the additional older participants resulting in a weakening of the correlation between lifetime noise exposure and age. We used six independent regression models (corrected for multiple comparisons), in which age, lifetime noise exposure, and high-frequency audiometric thresholds were used to predict measures of synaptopathy, with a focus on differential measures. The models for auditory brainstem responses, envelope-following responses, interaural phase discrimination, and the co-ordinate response measure of speech perception were not statistically significant. However, both age and noise exposure were significant predictors of performance on the digit triplet test of speech perception in noise, with greater noise exposure (unexpectedly) predicting better performance in the 80 dB sound pressure level (SPL) condition and greater age predicting better performance in the 40 dB SPL condition. Amplitude modulation detection thresholds were also significantly predicted by age, with older listeners performing better than younger listeners at 80 dB SPL. Overall, the results are inconsistent with the predicted effects of synaptopathy.

## Introduction

In a seminal study, Kujawa and Liberman (2009) described the phenomenon now known as cochlear synaptopathy. Mice were exposed to 100 dB sound pressure level (SPL) noise (8–16 kHz) for 2 hr, and it was subsequently found that up to 50% of synapses between inner hair cells and auditory nerve fibers were destroyed. This loss of synapses left absolute thresholds unaffected but was associated with a reduced Wave I of the suprathreshold auditory brainstem response (ABR) by 60% at 32 kHz and ∼30% at 12 kHz. Noise-induced synaptopathy has since been demonstrated in several rodent species (Hickox, Larsen, Heinz, Shinobu, & Whitton, 2017), and in a primate (macaque) model, although the noise levels required were higher, and the synapse loss lower, in the macaque compared with the mouse model (Valero et al., 2017). Furman, Kujawa, and Liberman (2013) provided evidence from single-unit recordings that the synapses with high-threshold, low- and medium-spontaneous-rate (SR) fibers were preferentially damaged during the process of noise-induced cochlear synaptopathy. This led to the *low-SR hypothesis* that suggests that the loss of synapses affects suprathreshold coding with minimal changes in responses close to threshold. Despite strong initial evidence in support of this hypothesis in animal models, there now exist a number of empirical findings that do not concord with this account of synaptopathy. In particular, Bourien et al. (2014) showed that low-SR fibers contribute little to the compound action potential (equivalent to ABR Wave I) in the gerbil. Hickox et al. (2017) provide a thorough overview of the different animal models, the exposures used, evidence found in support of the low-SR hypothesis, and those findings that are difficult to reconcile with this hypothesis.

The evidence for noise-induced cochlear synaptopathy in humans with normal audiometric thresholds is mixed. Stamper and Johnson (2015a) reported a decrease in ABR Wave I amplitude as a function of noise exposure from the previous 12 months. However, the groups of high and low noise exposure were imbalanced with regard to sex, and men tend to have smaller absolute amplitudes (Jerger & Hall, 1980) and more noise exposure than women. On reanalysis, a significant correlation was only found for women (Stamper & Johnson, 2015b). Bramhall, Konrad-Martin, McMillan, and Griest (2017) found that groups of highly noise-exposed veterans and nonveterans who use firearms have weaker ABR Wave I amplitudes than a group of nonfirearm users and a group of low-noise-exposed veterans. Liberman, Epstein, Cleveland, Wang, and Maison (2016) reported no difference in Wave I amplitude between groups of high- and low-noise-exposed listeners with clinically normal audiometric thresholds, but a large increase in the ratio of summating potential (SP) relative to the action potential in the high-risk group, driven largely by an increase in the SP. This ratio measure is proposed as a reliable measure of cochlear synaptopathy, although it remains unclear exactly how a loss of cochlear synapses would lead to an increase in the SP. In addition, the high-risk group had elevated audiometric thresholds at frequencies above 8 kHz, and it is unknown how the differences between the high-frequency hearing of the two groups might have affected the potentials. Liberman et al. (2016) also reported a deficit in speech-in-noise performance in the high-risk group at low sound levels (35 dB HL).

Grose, Buss, and Hall (2017) reported a reduction in another differential measure, the ABR Wave I/V ratio, in listeners who attended loud music events, although the authors found no behavioral effects related to this reduction. Valderrama et al. (2018) found a statistically significant reduction in ABR amplitude with increasing lifetime noise exposure in a group of listeners aged 29 to 55. These listeners had a mix of hearing profiles, and the degree of audiometric loss was not controlled for in the analyses. Although these two studies report evidence consistent with noise-induced cochlear synaptopathy in humans, the effect sizes were small, and neither significant finding would survive correction for multiple comparisons due to the number of outcome measures reported.

In contrast, a larger number of studies have found no evidence for a relation between noise exposure and electrophysiological responses (Fullbright, Le Prell, Griffiths, & Lobarinas, 2017; Grinn, Wiseman, Baker, & Le Prell, 2017; Guest, Munro, Prendergast, Howe, & Plack, 2017; Prendergast et al., 2017a, 2018; Smith et al., 2019; Spankovich et al., 2017), or between noise exposure and behavioral performance (Fullbright et al., 2017; Grose et al., 2017; Johannesen, Buzo, & Lopez-Poveda, 2019; Prendergast et al., 2017b; Smith et al., 2019; Yeend, Beach, Sharma, & Dillon, 2017), in cohorts of young adults with clinically normal audiograms. These cohorts differ in their demographics and in how noise exposure is quantified. It seems clear that, even if noise-induced cochlear synaptopathy does occur in humans with normal audiometric function, it cannot be easily measured and quantified. It is likely that humans are much less sensitive to noise damage than rodents; Dobie and Humes (2017) outline a number of caveats required when transposing sound exposures in laboratory rodent experiments into real-world human exposures.

An excellent overview and discussion of the literature on human studies of noise-induced cochlear synaptopathy highlights the common areas and differences between the various studies (Le Prell, 2019). Many studies focussed on participants in their 20 s and 30 s, with a small number explicitly testing older listeners. In most of the analyses, age was not explicitly controlled or modeled in the same analysis as the noise exposure estimate. Evidence from the mouse model suggests that aging is associated with synaptopathy in the absence of significant noise exposure (Sergeyenko, Lall, Liberman, & Kujawa, 2013), and there is growing histological evidence of age-related synaptopathy in humans (Makary et al., 2011; Viana et al., 2015; Wu et al., 2019), although it is not clear to what extent this reflects aging per se or the accumulative effects of noise exposure. Valderrama et al. (2018) did enter age and noise exposure into the same statistical model, and this model was only used to predict performance on a speech-in-noise task, not on electrophysiological proxies of cochlear synaptopathy. Age and noise exposure were not significant predictors of performance in the analysis reported (Valderrama et al., 2018). Grinn et al. (2017) also entered age and noise exposure into the same regression model to establish whether changes in audiometric function could be detected after recent noise exposure, although the age range was quite narrow (21–27 years). Johannesen et al. (2019) recently reported a study of ABR Wave I growth and speech reception thresholds as proxy measures for synaptopathy. They reported changes in Wave I ABR growth consistent with age-related, but not noise-induced, synaptopathy. There was also no evidence that Wave I ABR growth is related to speech-in-noise performance.

In our initial large-scale study on noise-induced cochlear synaptopathy in participants aged 18 to 35, age and noise exposure were highly correlated (*r* = .52; Prendergast et al., 2017a, 2017b). We observed some weak effects but, due to the nature of the cohort, were unable to establish if these changes were a result of noise exposure or of age. For the present article, we extended the upper age limit of the originally reported dataset and included participants with any noise exposure profile. This allowed us to model both age and noise exposure and to determine the extent to which they are able to account for the variance in the observed data. As we discussed when interpreting the findings of our initial study, it may be that a noise-induced loss of cochlear synapses is occurring in the young listeners, but the effects are too subtle to observe reliably. We speculated that a reduction in cochlear synapses due to noise exposure may be easier to observe via proxy measures in older listeners, as the noise exposure damage becomes convolved with age-related synaptopathy (Prendergast et al., 2017a).

For the current study, we obtained data from a cohort of listeners aged 37 to 60 on a subset of the tasks used in the initial study, described in Prendergast et al. (2017a, 2017b). We decided to include differential proxy measures of synaptopathy, which are designed to isolate the contributions of cochlear synaptopathy from between-subject variations in other aspects of auditory processing or anatomy (Plack et al., 2016). Age- and noise-induced cochlear synaptopathy is assumed to preferentially affect low-SR fibers, and therefore stimuli presented at a high sound level are most likely to be affected. Previously, we expected noise-induced synaptopathy to occur in the 3 to 6 kHz range, as this corresponds to the region in which noise-notches occur in audiograms, and hence, we used stimuli presented in low- and high-frequency regions to measure responses that were assumed to be less or more affected by the loss of synapses, respectively. However, as the age-related loss of synapses occurs uniformly across the cochlea length (Wu et al., 2019), for the analyses presented in this article, the differential measures were constructed across level. Therefore, each psychophysical task was performed at 40 and 80 dB SPL at 4 kHz. Each speech task was performed at these low and high sound levels (40 and 80 dB SPL). An envelope-following response (EFR) was measured at a single intensity (80 dB SPL) at 4 kHz. The ABR was normalized by scaling the Wave I amplitude relative to the Wave V amplitude in each listener. The following was the main research question: What are the relative contributions of age and noise exposure in predicting measures of cochlear synaptopathy?

## Methods

### Participants

Prendergast et al. (2017a) reported data from a cohort of 126 participants (75 women). For the extended data analysis, three of these participants were excluded, as there were no behavioral measures recorded due to participants leaving the study. The cohort consisted of 123 participants from the original data collection (*M* age = 23.11 years, *SD* = 4.26 years). An additional 33 older participants (24 women) were recruited (*M* age = 44.81 years, *SD* = 5.46 years) giving a final sample size of 156 (96 women). All participants had audiometric thresholds within the normal range up to 4 kHz (i.e., <25 dB HL), and at 8 kHz, all participants had audiometric thresholds of <35 dB HL. The procedures were approved by the University of Manchester Research Ethics Committee, and all participants gave informed consent (Project Number 14163).

### Noise Exposure

Lifetime noise exposure was estimated using the same methodology as described in Prendergast et al. (2017a, 2017b). Guest et al. (2018a) provide an exhaustive overview of a very similar approach, how to implement it, and the strengths and weaknesses of this methodology. The structured interview sought to quantify the number of hours, days, weeks, months, and years that a participant estimates placing themselves in an environment where the noise exposure exceeds 85 dBA. A formula was then used to compute the cumulative noise exposure over the lifetime. The raw noise immission units were log transformed to produce a normal distribution. Each logarithmic unit is equivalent to a factor of 10 in terms of lifetime exposure energy. A score of 0.5 represents relatively low noise exposure and could be achieved by going to a nightclub for 3 hr, once per month for 6 years. A score of 1.98 could be achieved by someone in their mid-30 s who has attended a nightclub/gig for 3 hr twice a week, every week, for the past 20 years. For the older listeners, their noise exposure scores may be made up of short-duration periods in their life when they were attending a large number of high-noise events, or a smaller number of events over a sustained period. Although this subjective recall approach has a number of limitations, the variance between subjects was large compared with the imprecision of the estimate. The approach has been shown to be able to discriminate between groups of listeners with and without tinnitus (Guest et al., 2017).

### Pure Tone Audiometry

Pure tone audiometry was performed for each ear separately at octave frequencies between 0.25 and 8 kHz in accordance with the British Society of Audiology (2011) recommended procedure. Thresholds were measured using VIASYS GSI-Arrow audiometers coupled to TDH-39 P supra-aural headphones, with MX41 cushions. High-frequency audiometry was also performed using a Creative E-MU 0202 USB soundcard. Sounds were played over Sennheiser HDA 200 circumaural headphones designed for high-frequency audiometry. The sound stimulus was a quarter-octave-wide band of noise centered at 16 kHz.

### Electrophysiological and Behavioral Measures

The specific and detailed implementations of the ABR and EFR are described in detail in Prendergast et al. (2017a) and the psychophysical and speech-in-noise tasks in Prendergast et al. (2017b). A summary of the stimuli, measures, and analyses is given here. For each task or recording, a single differential measure was used in the analysis to constrain the number of comparisons, with the exception of the EFR, which used the response from a single condition rather than a differential measure. In all cases, the justification for the choice of each differential measure is reported. For all of the tasks used, and for the differential measures constructed, the underlying assumption is that the low-SR account of how cochlear synaptopathy affects the physiology of the auditory system is accurate and therefore that coding of high-intensity stimuli is more compromised than that of low-intensity stimuli.

### Auditory Brainstem Response

All electroencephalography recordings were made in a single 2-hr session and used an ActiveTwo system (Biosemi, Amsterdam). Active electrodes were placed at the high forehead (Fz), the seventh cervical vertebra (C7), and the left and right mastoids (M1, M2). For the additional cohort of 33 participants, gold-plated active electrodes were used.

Stimuli were 100 -μs diotic clicks high-pass filtered at 1.5 kHz (using a fourth order Butterworth filter) and presented binaurally in alternating polarity. Because of the low-pass characteristic of the ER3A inserts, the stimulus delivered to the ear had a restricted bandwidth with a spectral plateau from about 1.5 to 4 kHz. Click levels were 100 dB peSPL (measured at the output of the inserts using an IEC711 2-cc coupler). Presentation rate was 11 clicks/s with 7,480 presentations in total. Clicks were presented in 18-s blocks. The 198 clicks presented within a block were not jittered, but the timing of the blocks was jittered.

Differential voltage waveforms were created using Fz-M1 and Fz-M2, and these two montages were then averaged to give the final ABR waveform. Peak-to-trough amplitudes were automatically extracted from the ABR waveform using custom software that is described fully in Prendergast et al (2017a). The differential measure used for the analyses presented here was the Wave I/Wave V amplitude ratio.

### Envelope-Following Response

Two contiguous acquisitions were made, and in each acquisition, four tones were presented simultaneously, with a low-frequency tone (240–285 Hz) and a low-frequency tone (240–285 Hz) transposed to 4 kHz (Bernstein & Trahiotis, 2002) presented to each ear. For one acquisition, the left ear received a 255 Hz pure tone and a 240 Hz transposed tone, and the right ear received a 270 Hz pure tone and a 285 Hz transposed tone. For the other acquisition, the left ear received a 285 Hz pure tone and a 255 Hz transposed tone, and the right ear received a 240 Hz pure tone and a 270 Hz transposed tone. Stimuli were 220 ms in duration (including 10 ms ramps) and presented at 80 dB SPL. Each stimulus was presented 4,000 times in alternating polarity (2,000 repetitions for each polarity) with an interstimulus interval randomly selected within the range 85 to 95 ms.

The montage used for the analysis was Fz-C7. For each polarity, sweeps were maintained for further analysis if their root-mean-square power was no greater than two standard deviations above the mean for the recording. Included sweeps were averaged in the time domain to produce an average for each polarity. These averages were summed to produce a waveform that contains the EFR for the high-frequency region and also subtracted to produce a waveform that emphasizes the fine structure frequency-following response for the low-frequency region. The fine structure frequency-following response was not included in the analyses presented in this article. A 200-ms window was used for the analysis, which began 10 ms after stimulus onset. Once the EFR was extracted from the raw data, the four responses (two from each acquisition) were expressed as signal-to-noise ratios (SNRs), the average of these converted into dB (20log10[SNR]), and this was used as the dependent variable for the EFR measure.

### Psychophysical Tasks

All stimuli were presented using a Creative E-MU 0202 USB soundcard and Sennheiser HD650 circumaural headphones and digitized in the creation and presentation stages at a sampling frequency of 48 kHz. Interaural phase difference (IPD) and amplitude modulation detection (AMD) thresholds were measured using a two-down, one-up adaptive staircase. Thresholds were estimated using the average of the final 10 reversals for each run and across three runs. Both tasks were performed at a low- and high-frequency region (255 Hz and 4 kHz, respectively) and a low and high sound intensity (40 and 80 dB SPL, respectively). For the analyses presented in this article, only the 4-kHz conditions were used, and the differential measure constructed was 80 dB SPL to 40 dB SPL. The differential measure is expressed in logarithmic units, with the sign denoting the direction of the difference (i.e., a positive value indicating worse performance in the high-intensity condition).

For the IPD task, an AAAA versus ABAB paradigm was used. Tones were 300 ms in duration (including 50 ms raise-cosine ramps). The stimulus was a transposed tone, consisting of a 4-kHz tonal carrier modulated by a half-wave rectified, and low-pass filtered, 255-Hz pure tone. This processing allows the neural representation of the high-frequency stimulus envelope to mimic the neural representation of the low-frequency pure tone once it has undergone internal peripheral processing (which can be modeled as a rectification and subsequent low-pass filter; Bernstein & Trahiotis, 2002). The IPD for the standard stimulus (A) was always zero. For the comparison stimulus (B), the IPD for the pure tone or pure-tone modulator was varied adaptively. The starting difference was 30°, generated by advancing the phase for the right-ear signal. The IPD was varied geometrically using an initial step size of a factor of 1.56 and a final step size factor of 1.25. The maximum IPD for stimulus B was restricted to 90°. If the maximum difference was reached, the difference remained fixed until two correct responses were given consecutively. For the high-frequency condition, low-pass pink noise was added to mask combination tones. The cutoff frequency of the noise band was 2.5 kHz, and the spectrum level at 1 kHz was 40 dB below the signal level.

For the AMD task, a three-alternative forced-choice paradigm was used. Stimuli were 200 ms in duration. The carrier was a 4-kHz pure tone that was sinusoidally amplitude modulated at 25 Hz. The root mean square energy was equated across intervals. The starting modulation depth was 50%, and this was then geometrically varied according to a two-down one-up track with an initial step size factor of 1.56 and a final step size factor of 1.25. There was a 500-ms interstimulus interval between each of the three tones.

### Speech Tasks

The co-ordinate response measure (CRM) was used (Bolia, Nelson, Ericson, & Simpson, 2000). The participant was presented with a number of speech utterances of the structure “*Ready* < *call sign* > *go to* < *color* > <*number* > *now*,” in which there were eight unique call signs, four different colors (Blue, Red, White, Green), and the number was in the range 1 to 4. The participant’s call sign was always *Baron*. Two maskers were presented simultaneously, which were always different speakers and different call signs, although the color and number could match that of the target. The CRM was performed at two sound levels (40 and 80 dB SPL) that defined the level of the combined masker stimuli. The target was diotic. For the additional data collection on the cohort of 33, maskers were only ever presented diotically, whereas in the initial data collection, there was also a condition in which the maskers were spatially offset. For each individual, a cumulative Gaussian was fitted to the data to model the distribution and to allow the SNR to be interpolated for achieving 25% correct identification. The summary metric used was the difference in SNR between thresholds for the 40 and 80 dB SPL conditions (80–40).

The digit triplet test (DTT; Smits, Kapteyn, & Houtgast, 2004) was also used, in which participants were required to identify three spoken digits (in the range 1–9) presented sequentially in a speech-shaped background noise (0–10 kHz bandwidth). The noise was fixed at each of two levels (40 and 80 dB SPL), while the sound level of the spoken digits was varied. A method of constant stimuli was used, with six repetitions at each of eight SNRs presented per block of trials. Three randomly interleaved blocks were presented for each sound intensity, resulting in 18 presentations for each SPL-SNR combination. The overall percent correct responses were calculated for each condition. The SNRs used were −24 to −3 dB in steps of 3 dB. Cumulative Gaussians were fit to the data and, for both presentation levels, the SNR corresponding to 25% correct identification was taken forward into the statistical analyses. This was because in Prendergast et al. (2017b), we reported thresholds for 25%, 50%, and 75% correct response criteria, and though the relation with noise exposure was weak, it was strongest for the most challenging conditions. The summary metric used was the difference in SNR between the thresholds for 40 and 80 dB SPL (80–40).

### Statistical Analyses

To answer the two research questions, six multiple regression models were run with an adjusted alpha of .008 (to account for the six individual models). Age, noise exposure, and 16-kHz audiometric threshold were entered as predictors. Noise exposure was entered, as this was previously the main predictor of interest in our studies of noise-induced cochlear synaptopathy. Age was included to evaluate if it accounted for any variance independently of noise exposure. We also included 16-kHz audiometric thresholds, as these have been proposed to be an early marker of noise-induced synaptopathy, and in a number of studies showing differences in ABR amplitudes as a function of exposure group, there are often audiometric differences in the high-frequency region (e.g., Grose et al., 2017; Liberman et al., 2016). Our inclusion of this measure is based on the rationale that the responses, specifically the ABR amplitudes, may be affected by high-frequency audiometric hearing loss. Including this measure as a predictor in the regression analyses allowed us to control for these hearing thresholds when assessing the effect of age and noise exposure on performance.

The models were used to predict the dependent variables highlighted in the methods section (ABR: Wave I/V ratio; EFR: 4 kHz SNR; IPD: 80–40 dB SPL presented at 4 kHz; AMD: 80–40 dB SPL presented at 4 kHz; CRM: 80–40 dB SPL; DTT: 80–40 dB SPL). All analyses were performed using the linear regression model in SPSS (IBM Corp, Version 23). The aim of the extension to the cohort was to provide additional data within which age and noise exposure were not as strongly related as in the original cohort. We would then be better able to disentangle the two parameters.

## Results

### Noise Exposure

**Figure 1.**
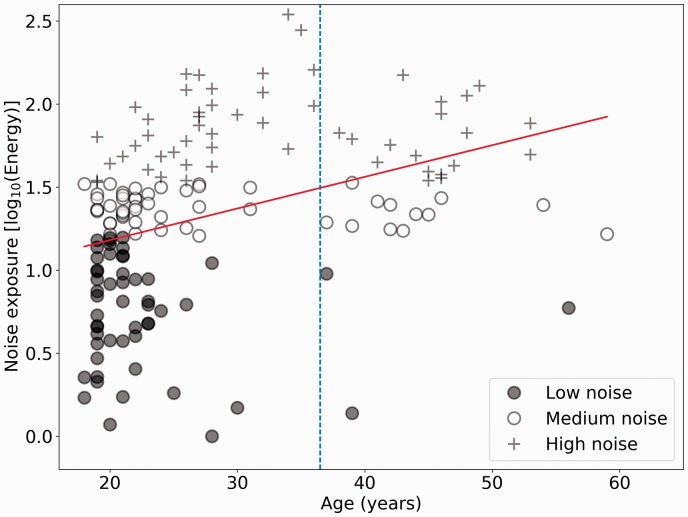
Noise exposure scores as a function of age for 156 participants. The least squares regression line is also shown in red. The lower, middle, and upper thirds of the noise exposure cohort are plotted in different symbols. The age cutoff for the two cohorts is shown by the dashed vertical line.

Figure 1 shows estimated lifetime noise exposure scores for all 156 participants as a function of age. The two cohorts are identified by the dashed vertical line. Note that the *y* axis is a logarithmic scale with respect to energy: The individuals with the highest exposures have about 300 times the lifetime exposure energy of those with the lowest exposures. Average lifetime noise exposure for the 123 younger listeners is 1.27 (*SD* = 0.53), while the average exposure for the older cohort is 1.52 (*SD* = 0.40). The Spearman correlation between noise exposure and age is 0.50 (*p* = 4.16e-11) for the full cohort (*N* = 156) and for the older group of 33 listeners alone is 0.24 (*p* = .17).

### Audiograms

**Figure 2.**
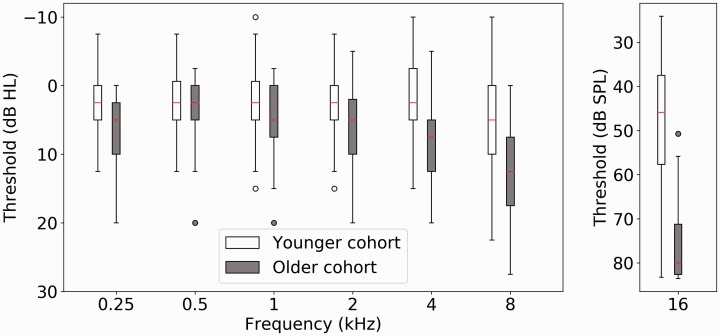
Boxplots are shown in dB HL for the standard audiometric frequencies (0.25–8 kHz) and in dB SPL for 16 kHz. The line denotes the median and the box length the interquartile range. Whiskers show the extent of the data with outliers (defined as ±1.5 × IQR) plotted as individual points. The open boxes denote thresholds from the younger cohort and the filled boxes the older listeners. SPL = sound pressure level.

Figure 2 shows audiometric and 16 kHz thresholds for the younger cohort of 123 listeners, and the older cohort consisting of 33 listeners, averaged between the ears. The median score (shown in red in each of the boxes) for the older, smaller, group of participants is greater than that of the younger cohort at each frequency tested. The boxes, denoting the central 50% of data points, are markedly displaced between the groups at 4 and 8 kHz. This displacement is more pronounced at 16 kHz, where many of the older participants were constrained by the maximum permitted output of 85 dB SPL.

### Electrophysiology

**Figure 3.**
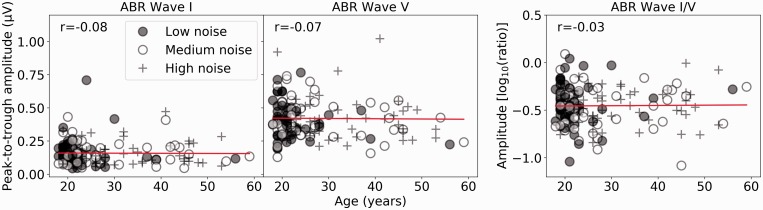
ABR responses, with Wave I amplitude, Wave V amplitude, and the Wave I/V ratio plotted as a function of age in the left, central, and right panels, respectively. The different thirds of the noise exposure cohort are plotted in different symbols. Spearman correlation coefficients are reported. ABR = auditory brainstem response.

**Figure 4.**
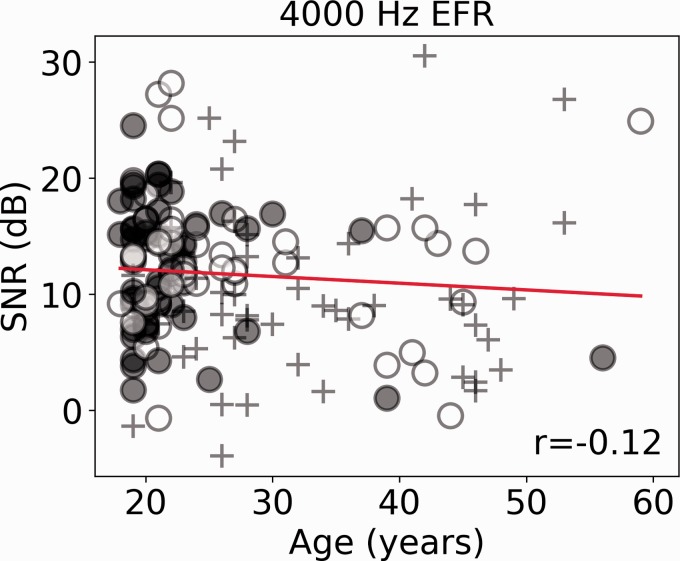
The EFR response is plotted as a function of age. Solid (red) lines show the linear regression line, and the different thirds of the noise exposure cohort are plotted in different symbols. The Spearman correlation coefficient is reported. SNR = signal-to-noise ratio; EFR = envelope-following response.

Figure 3 shows the ABR Wave I, V, and I/V amplitudes in response to a 100 dB peSPL click for the full cohort of participants as a function of age, with the different symbols representing the lower (filled circles), middle (open circles), and upper (plus signs) thirds of the noise exposure cohort. The least squares regression lines are plotted, and the Spearman correlation coefficients are shown. Figure 4 shows the EFR as a function of age. The regression line indicates only slight deviations from a random relation between noise exposure and the EFR. For both electrophysiological responses, there appears to be nothing substantially different in the responses of the older listeners; their data are not qualitatively different from those of the younger cohort. This is despite evidence that older participants have fewer synaptic connections than younger people (Viana et al., 2015; Wu et al., 2019), and in this cohort (as shown in Figure 1), the older listeners are likely to have had greater lifetime noise exposure than the younger listeners. Therefore, one would predict the size of these responses to be reduced in older listeners relative to the younger listeners. The fact that they are not indicates that these measures are not sensitive enough to pick up subtle changes in auditory coding. What can be seen clearly in Figures 3 and 4 is the large variability in response amplitude seen for participants aged 18 to 20. As this between-subject variability is so high, a large sample size would be needed to estimate the effect of age on these electrophysiological measures.

### Psychophysics

**Figure 5.**
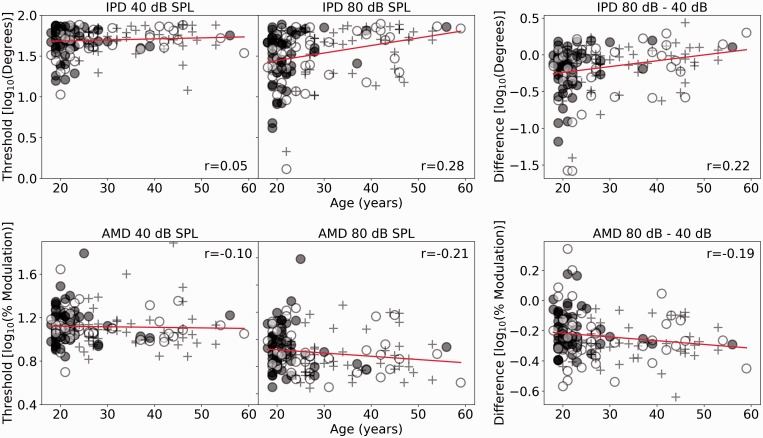
The upper row shows IPD responses, and the lower row shows AMD response. For both rows, 40 dB SPL, 80 dB SPL, and the differential measures are plotted as a function of age in the left, central, and right panels, respectively. Solid (red) lines show linear regressions. Spearman correlation coefficients are reported. IPD = interaural phase difference; SPL = sound pressure level; AMD = amplitude modulation detection.

Figure 5 shows the psychophysical thresholds for the full cohort of participants as a function of age, with the symbols differentiating the lower, middle, and upper thirds of the noise exposure cohort. The least squares regression lines are plotted, and the Spearman correlation coefficients are shown. For the AMD task, the older listeners tend to perform better than the younger listeners at both sound levels. For the IPD task, performance appears relatively stable with age for the low-level condition. As for the electrophysiological measures shown in Figures 3 and 4, the between-subject variability of the responses at a single age on the continuum appears to be as large as any variability in the response across different ages.

### Speech in Noise

**Figure 6.**
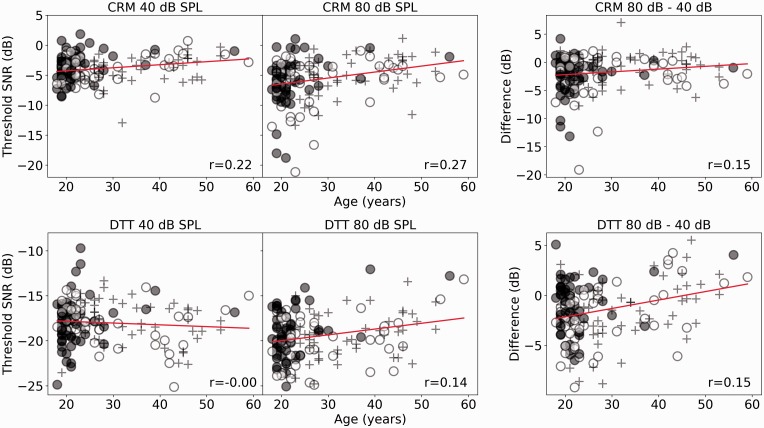
The upper row shows SNRs corresponding to the 25% correct-response criterion for the CRM task, and the lower row shows SNRs corresponding to the 25% correct-response criterion for the DTT task. For both rows, the 40 dB, 80 dB, and the differential measures are plotted as a function of age in the left, central, and right panels, respectively. Solid (red) lines show linear regressions. Spearman correlation coefficients are reported. CRM = co-ordinate response measure; SPL = sound pressure level; DTT = digit triplet test; SNR = signal-to-noise ratio.

Figure 6 shows the CRM and DTT thresholds for the full cohort of participants as a function of age, with the symbols differentiating the lower, middle, and upper thirds of the noise exposure cohort. The least squares regression lines are plotted, and the Spearman correlation coefficients are shown. For the differential measure, a negative value indicates that listeners performed better in the 80 dB condition compared with the 40 dB condition. For the CRM task, the older listeners, on average, show higher thresholds than the younger listeners at both intensities, which result in the *R*^2^ for the differential measure being lower than that of both the individual conditions. For the DTT, performance across the continuum is largely equivalent at 40 dB SPL, and older participants tend to show higher thresholds at 80 dB SPL.

### Multiple Regression Models

Table 1 shows the *R*^2^ and significance levels for the six regression models. The models for the DTT and AMD tasks reach statistical significance. Further inspection of the DTT model indicates that both age and noise exposure are significant predictors of the differential measure (standardized beta coefficients of .441 and −.288, respectively, and *p* values of .0001 and .001, respectively). Surprisingly, the predictors of age and noise exposure have opposite effects on the differential measure. Increasing age is associated with an increase in the differential measure (consistent with increasing synaptopathy), and increasing noise exposure is associated with a decrease in the differential measure (inconsistent with increasing synaptopathy). This is based on the assumption that listeners with cochlear synaptopathy will perform less well in the high-intensity, 80 dB SPL, condition compared with the low-intensity, 40 dB SPL, condition. 16-kHz audiometric thresholds are not a significant predictor. One concern is that responses in the 40 dB condition are affected by audiometric threshold and that listeners with poorer hearing perform less well on the task. However, the Spearman correlation between the 40-dB DTT threshold and the between ear average of pure tone thresholds at 4 and 8 kHz was −0.152 (*p* = .058). The negative correlation indicates that listeners with higher absolute thresholds perform slightly better on the task (yield a lower SNR value for 25% correct). A second concern was that, due to issues of collinearity between the predictor variables, the individual coefficients could be an inaccurate description of the underlying relations in the data. However, for the three regression predictors, the largest variance inflation factor was 2.03, which indicates multicollinearity is not a concern, as harmful multicollinearity is considered to be present only when this value reaches 10 (Marquardt, 1970).

**Table 1.** Outcomes of the Six Regression Models Are Shown.

Dependent variable	Adjusted *R*^2^	*F*(*df*)	*p* value
ABR (Wave I/V)	.002	1.085 (3, 140)	.358
EFR (4 kHz SNR)	−.004	0.801 (3, 143)	.495
IPD (80–40 dB SPL)	.034	2.738 (3, 146)	.046
AMD (80–40 dB SPL)	.593	72.362 (3, 144)	<.001*
CRM (80–40 dB SPL)	.011	1.558 (3, 146)	.202
DTT (80–40 dB SPL)	.136	8.788 (3, 146)	<.001*

*Note*. Age, noise exposure, and high-frequency hearing thresholds were entered into the model. Note that adjusted *R*^2^ can be negative in weak regression models. ABR = auditory brainstem response; EFR = envelope-following response; SNR = signal-to-noise ratio; IPD = interaural phase difference; SPL = sound pressure level; AMD = amplitude modulation detection; CRM = co-ordinate response measure; DTT = digit triplet test.

* denotes a statistically significant model (alpha = .008).

**Figure 7.**
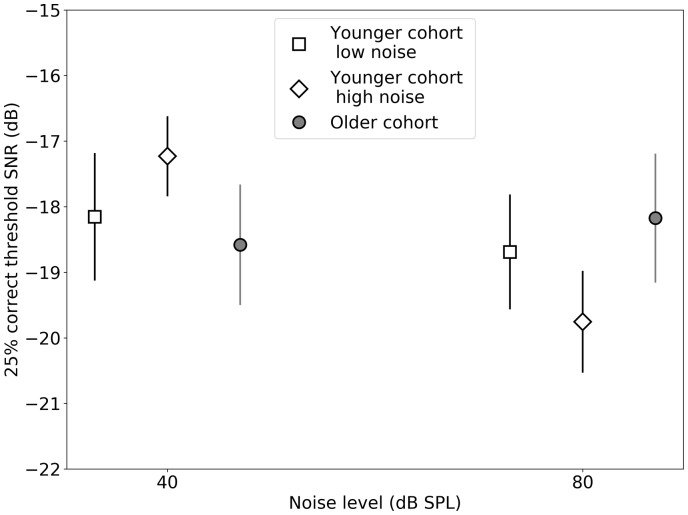
Mean DTT thresholds (and 95% confidence intervals) are shown for groups of low-noise, high-noise, and older listeners (open square, open diamond, and filled circle, respectively). SNR = signal-to-noise ratio; SPL = sound pressure level.

Figure 7 provides a visual illustration of how the predictors and the individual conditions of the DTT interact. Thresholds are plotted for a group of 32 lowest noise-exposed listeners, the 32 highest noise-exposed listeners (both groups first presented in Prendergast et al., 2017b, and therefore both from the younger listeners in this extended cohort), and the 33 older participants from the present study. Age and noise exposure interact across these groups, with the low-noise group also being the youngest, and the older cohort being closer in noise exposure to the high-noise rather than the low-noise group. The older group does not contain any participants who engaged in critical listening by virtue of their job (i.e., they do not work as professional musicians, sound engineers/technicians). The difference between the 40 dB and 80 dB conditions is small (∼0.5 dB) for the low-noise and older groups of listeners. The difference for the high-noise group is larger (∼2.5 dB), and this group performed better in the 80 dB condition versus the 40 dB condition.

The model including age, noise exposure, and high-frequency audiometric thresholds is also a significant predictor of performance for the AMD differential measure. Further inspection of the model parameters indicates that only age is a significant predictor of performance (standardized beta coefficient of −.744, *p* < .001), with older listeners performing better in the 80 dB condition compared with the 40 dB condition relative to the younger listeners. There is again the concern that audiometric threshold may account for the differences across the group, even though these thresholds are all within clinically normal limits. The Spearman correlation coefficient between the AMD differential measure and 4 kHz audiometric threshold is −0.34 (*p* < .001), indicating that as audiometric threshold gets worse, performance on the AMD task improves. This issue is studied in more detail in the following section.

### Exploratory Analyses

Two additional exploratory analyses were performed to understand better what factors may account for the significant regression model of the DTT differential measure. First, the variables of age and noise exposure were centered by demeaning and an interaction term was used in a model with age and noise exposure to examine whether any independent variance was explained by this term. The model is significant, adjusted *R*^2 ^= .147, *F*(3, 152) = 9.88, *p* < .05e-4, with age and noise exposure both still significant predictors for the DTT differential measure (beta = .395 and −.296, *p* < .03e-4 and *p* < .03e-2, respectively). The interaction term is not a significant predictor of performance (beta = .035, *p* = .646). The model is also significant for the AMD task, adjusted *R*^2 ^= .209, *F*(3, 150) = 14.479, *p* < .05e-4, with age still the only significant predictor of performance (standardized beta = −.469, *p* < .05e-4). Noise exposure and the interaction term are not significant predictors of performance.

Prendergast et al. (2017b) evaluated the relation between musical experience (number of years over which a musical instrument was played regularly) and behavioral performance. Additional regression models were tested, one with age and musical experience used as predictors and one with noise exposure and musical experience used as predictors. The model containing age and musical experience significantly predicts the DTT differential measure, *F*(2, 153) = 8.398, *p* = .0003, but musical experience itself is not a significant predictor (standardized beta = −.118, *p* = .127). The model with noise exposure and musical experience is not a significant predictor of the differential DTT measure, *F*(2, 153) = 2.454, *p* = .089. The same is seen for the AMD models, with musical experience not modifying the outcome previously seen, in which age is the only significant predictor of performance.

#### The role of audiometric thresholds

Although the audiometric criteria for inclusion required participants to have clinically normal hearing up to 4 kHz, this still leaves 30 dB of variability. Many studies, including ones from our laboratory, equate the hearing of listeners in this way and often overlook the possibility that performance on tasks and electrophysiological measures are still dependent on audiometric thresholds in the standard range. To test this, we reran the regression models reported in Table 1, but for each task, the 16-kHz hearing thresholds were replaced with the thresholds at a tone frequency within the normal range. For each measure, the audiometric thresholds used were those which correlated most strongly with the dependent variable. This analysis was prompted by the fact that the older listeners appear to have worse hearing in the standard audiometric range than the younger listeners (see Figure 2), and this could therefore mask any differences due to noise exposure or age.

By selecting the audiometric frequency that most strongly correlates with the dependent variable, we are ensuring that the model has the best chance possible of highlighting the role of audiometric thresholds. This is particularly important with respect to the initial DTT model as it is now as easy as possible for this second model to cast doubt on the veracity of the initial model that pointed to age and noise exposure as independent predictors. It is also important for the AMD model to establish whether this task does vary as a function of age, independent of audiometric thresholds. As the audiometric threshold was chosen for the reason of its high correlation with the dependent variable, no inference should be made regarding any models in which only audiometric threshold is a significant predictor of performance; the crucial aspect is whether including audiometric threshold changes the predictive value of age or noise exposure relative to the initial model.

The thresholds entered for each model and the correlation between audiometric threshold and the dependent variable are as follows: ABR and 1 kHz threshold, *r* = −.29; EFR and 250 Hz threshold, *r* = −.07; IPD and 4 kHz threshold, *r* = .34; AMD and 4 kHz threshold, *r* = −.34; CRM and 2 kHz threshold, *r* = .16; and DTT and 500 Hz threshold, *r* = −.22. If the same alpha is used as for the previous models (0.008) despite the fact that these are now additional tests, then the relation between audiometric threshold and the dependent variable is significant for the ABR, IPD and DTT measures.

Table 2 shows the adjusted *R*^2^ values for the regression models. If these are compared with those in Table 1, it is clear that for each measure, except the AMD differential measure, the model including audiometric thresholds from a single frequency within the standard clinical range is a better estimator of the data than a model including 16-kHz thresholds. Two of the six models are not statistically significant: EFR and CRM. For the two measures that were previously nonsignificant (ABR and IPD), age and noise exposure are not significant predictors of the differential measure, and only the audiometric threshold significantly predicts performance: ABR, 1-kHz threshold standardized beta = −.306, *p* < .001; IPD, 4-kHz threshold standardized beta = .298, *p* = .001.

**Table 2.** The Outcomes of the Six Regression Models in Which 16-kHz Thresholds Were Replaced by Hearing Thresholds at a Frequency From the Standard Audiometric Range (0.25–8 kHz) Are Shown.

Dependent variable	Audiometric threshold entered	Adjusted *R*^2^	*F*(*df*)	*p* value
ABR (Wave I/V)	1000 Hz	.077	5.104 (3, 146)	.002*
EFR (4 kHz SNR)	250 Hz	−.004	0.815 (3, 148)	.488
IPD (80–40 dB SPL)	4000 Hz	.110	7.378 (3, 152)	<.001*
AMD (80–40 dB SPL)	4000 Hz	.222	15.518 (3, 150)	<.001*
CRM (80–40 dB SPL)	1000 Hz	.027	2.405 (3, 152)	.070
DTT (80–40 dB SPL)	500 Hz	.219	15.45 (3, 152)	<.001*

*Note*. Age, noise exposure, and hearing thresholds were entered into the model. Note that adjusted *R*^2^ can be negative in weak regression models. ABR = auditory brainstem response; EFR = envelope-following response; SNR = signal-to-noise ratio; IPD = interaural phase difference; SPL = sound pressure level; AMD = amplitude modulation detection; CRM = co-ordinate response measure; DTT = digit triplet test.

* denotes a statistically significant model (alpha = .008).

The initial DTT model, which included 16-kHz thresholds, shows that all three predictor variables significantly predict the differential measure of performance. For the new model, including 500-Hz thresholds instead, again all three parameters are significant predictors of performance: noise exposure standardized beta = −.285, *p* < .001; age standardized beta = .445, *p* < .001; and 500-Hz thresholds standardized beta = −.272, *p* < .001.

The initial AMD model that included 16-kHz thresholds is highly significant, and the only statistically significant independent predictor is age. For the new model, including 4-kHz thresholds, again age is the only significant predictor of performance: standardized beta = −4.732, *p* < .001.

#### Relations of measures to ABR Wave I

ABR Wave I amplitude is a proxy of the degree of cochlear synaptopathy present (Grose et al., 2017; Kujawa & Liberman, 2009; Sergeyenko et al., 2013). Hence, it is informative to use this to test the hypothesis that synaptopathy is related to other measures of auditory function. A similar approach has been used by Johannesen et al. (2019) to estimate the effect synaptopathy (as estimated by ABR amplitude) has on a number of measures of auditory function.

For this analysis, two regression models were run for each of five dependent variables. For the first model, ABR Wave I amplitude was entered as a single predictor variable. For the second model, ABR Wave I amplitude was entered, with age, noise exposure, and high-frequency audiometry entered in a stepwise, iterative scheme. This exploratory analysis allows an investigation of whether the objective measure of auditory nerve function alone is able to predict auditory coding fidelity on a number of measures. Table 3 shows the results of these analyses. ABR Wave I amplitude is not a significant predictor of performance on any of the five measures tested. For the stepwise model that sequentially and iteratively introduced age, noise exposure, and high-frequency audiometric thresholds, these analyses yield no new information compared with the models in Table 1; the DTT is significant due to age and noise exposure interacting in different directions and performance on the AMD task is predicted only by age.

**Table 3.** Outcomes of the Analyses in Which Model 1 Used the ABR Wave I Amplitude to Predict the Dependent Variables and Model 2 Included Wave I Amplitude as a Constant and Added Age, Noise Exposure, and High-Frequency Hearing as Determined by a Stepwise Procedure.

Dependent variable	Adjusted *R*^2^		*F*(*df*)		*p* value	
	Model 1	Model 2	Model 1	Model 2	Model 1	Model 2
EFR (4 kHz SNR)	.034	–	5.955 (1, 141)	–	.016	–
IPD (80–40 dB SPL)	−.002	.037	0.767 (1, 142)	3.710(2, 141)	.383	.027
AMD (80–40 dB SPL)	−.004	.579	0.393 (1, 140)	97.833(2, 141)	.532	<.001*
CRM (80–40 dB SPL)	.018	–	3.661 (1, 141)	–	.058	–
DTT (80–40 dB SPL)	.000	.142	0.996 (1, 142)	8.883(3, 140)	.320	<.001*

*Note*. Adjusted *R*^2^ can be negative in weak regression models. EFR = envelope-following response; SNR = signal-to-noise ratio; IPD = interaural phase difference; SPL = sound pressure level; AMD = amplitude modulation detection; CRM = co-ordinate response measure; DTT = digit triplet test.

* denotes a statistically significant model (alpha = .008).

## Discussion

### No Evidence for Age- or Noise-Induced Cochlear Synaptopathy

The following was the research question posed: What are the relative contributions of age and noise exposure in predicting measures of cochlear synaptopathy? To answer this question, we extended the age range of the original cohort. The addition of 33 older listeners to the cohort reduced the correlation between age and noise exposure, providing us with greater capacity to dissociate the two parameters.

Reduced electrophysiological responses of auditory function are most commonly used as a proxy measure for cochlear synaptopathy in both animal and human studies (see Bramhall et al., 2019; Hickox et al., 2017; Le Prell 2019 for comprehensive overviews). With the extended age range in the current study, we found no evidence that age or noise exposure predicts the differential response measures of the ABR, or the EFR amplitudes.

The only measures that result in a statistically significant regression model are the DTT and AMD. For the DTT, noise exposure is a significant predictor of performance, but the pattern of results is not consistent with the low-SR synaptopathy hypothesis. Noise-exposed participants performed relatively better in the 80 dB condition than in the 40 dB condition, whereas the low-SR hypothesis of noise-induced synaptic loss would predict performance to be impaired in the 80 dB condition due to the loss of high-threshold fiber terminals. Age also has a significant effect on the DTT measure, with older participants performing slightly better than younger listeners at 40 dB but worse in the 80 dB condition. For the AMD task, detection thresholds improved with increasing age. Neither of these models appear to be confounded by differences in audiometric threshold, nor do they support the simple hypothesis, suggested by the low-SR hypothesis of synaptic loss, that noise-induced and age-related synaptopathy will result in a reduction in performance for high-intensity sounds.

In summary, our findings are consistent with studies from our laboratory using distinct cohorts (Guest et al, 2017; Guest, Munro, Prendergast, Millman, & Plack, 2018b; Prendergast et al., 2017a, 2017b), and those from other research groups (Fullbright et al., 2017; Grinn et al., 2017; Smith et al., 2019; Spankovich et al., 2017; Yeend et al., 2017), suggesting that there is little effect of recreational noise exposure on auditory function for individuals with clinically normal audiometric thresholds.

The model that significantly accounts for variance in the DTT differential measure indicates that age contributes to the model more than noise exposure. Furthermore, these two parameters predict opposite effects on the differential measure. Increasing age results in a larger differential measure, with performance in the 80 dB condition relatively worse than that in the 40 dB condition, whereas noise exposure predicts the opposite relation, with increasing noise exposure predicting performance to be relatively better in the 80 dB condition. This is difficult to reconcile as, if a person still engages in exposure to loud recreational music as they age, then these two behaviors, according to the model presented in this article, act in opposite directions on the differential measure of performance. It should be noted that the effect of noise exposure on DTT performance was reported in Prendergast et al. (2017b), and as many of the participants are the same as in the current analysis, it is not particularly surprising that this relation persists. What is of interest is the fact that the model identifies noise exposure and age as independent predictors of performance. Also, Prendergast et al. (2017a, 2017b) reported weak but significant relations (uncorrected) for differential measures of IPD, AMD, CRM, and EFR and noise exposure. However, for the analyses presented here, in which many participants are the same, these relations are not significant.

As the human auditory system ages, it is well established that loss of outer hair cell function leads to poorer hearing (Lee, 2013), and there is evidence for additional age-related neural deficits, particularly affecting temporal processing (e.g., Füllgrabe, Moore, & Stone, 2015). Furthermore, aging has been shown to lead to a loss of cochlear synapses in animals (Parthasarathy & Kujawa, 2018; Sergeyenko et al., 2013) and in human temporal bones examined postmortem (Viana et al., 2015; Wu et al., 2019). However, the temporal bone studies published to date have little information about the noise exposure of the human listeners. A crucial issue that has not been resolved is the extent to which age-related deficits in humans reflect the effects of age per se or the cumulative effects of noise exposure.

The data presented in this article underline the difficulty inherent in dissociating the effects of aging from those of noise exposure. Even if a reliable proxy measure for a loss of cochlear synapses were found, it is difficult to see how this would translate into a more accurate assessment of the causes of a patient’s auditory disability. For instance, if a person performs worse than expected (for their age-relevant norms) on the DTT task at 80 dB, is this because they are in the tail of the distribution for age-related changes in hearing, or because they have additional deficits due to noise exposure? For a second listener who is more noise exposed, the model would predict their performance to be better than the first person, but there is variability in the onset of age-related changes, and there are individual differences in the susceptibility to damage from noise exposure (Henderson, Subramaniam, & Boettcher, 1993).

One option to try to disentangle these two factors might be to model the effects of different hearing profiles, degrees, and types of loss (i.e., outer hair cell, inner hair cell, synaptic connection, and fiber group). Difficulties remain with such an approach, as they do not prevent age and noise exposure covarying in the populations under examination, but they perhaps allow researchers to make predictions about the expected pattern of performance on various measures, given that the low-SR hypothesis now seems inadequate to explain how noise-induced cochlear synaptopathy might manifest itself in humans (Carney, 2018; Verhulst, Altoè, & Vasilkov, 2018; Verhulst, Jagadeesh, Mauermann, & Ernst, 2016). Another effective approach would be to observe changes longitudinally, but such studies are time-consuming and cost-intensive to implement.

### What Can the DTT Tell Us About Cochlear Synapses?

It remains unclear from the analysis presented here what is driving the significance of the DTT model. The highest noise-exposed listeners performed differently on the two conditions of the task (40 and 80 dB SPL) compared with less noise-exposed participants. However, caution is needed when considering this finding. Valderrama et al. (2018) note that the speech-in-noise performance of their listeners was related to attentional factors. Many of the highest noise-exposed listeners in our study worked in the music industry in some way and could be described as critical listeners; they regularly focus their attention on complex soundscapes that are presented at high intensities. Therefore, they may be better trained at performing tasks in which the sound level is high. They may also be better at developing strategies for the task and may be more motivated to engage with the task, that is, maintain their attention. It would be of value to find a group of noise-exposed listeners who do not listen critically to the form and character of the noise they are exposed to.

The significance of the DTT model may be able to inform us of the type of speech task we should use as we continue to investigate the effect of noise on the auditory system. The DTT is clearly sensitive to a regression model with age and noise exposure as predictors, whereas the CRM task, in which the maskers are speech rather than steady noise, is not. Furthermore, we focussed here on the threshold estimated to correspond to 25% correct on the psychometric function. This was motivated by the fact that in Prendergast et al. (2017b), the differential measure for the DTT was not significant for the 75% correct point on the psychometric function and was strongest (though still only weak) for the SNR at which 25% correct responses were observed. We speculated that in healthy, normal hearing listeners, it may be necessary to challenge the coding resources to see subtle differences across people. Conversely, it may be that making the task more difficult, that is, using the 25% point on the psychometric function, increases the reliance on nonauditory factors, such as attention. Listeners who are engaged in the task, or who are experienced in listening in such a way, may perform better for reasons unrelated to auditory coding.

### The Definition of Normal Hearing in Experimental Research

The exploratory analysis in which audiometric thresholds from the standard range were entered into the regression models adds a number of important discussion points to the work presented in this article: (a) that the significant DTT and AMD models initially seen do not appear to be dependent on any difference in audiometric thresholds within the standard range and (b) that although a cohort can have clinically normal hearing, they should not necessarily be considered to have equivalent hearing, as their performance on a task may still be related to their audiometric thresholds (performance on the IPD task was predicted by 4 kHz audiometric thresholds). Both of these points have implications for how future studies of subclinical listening deficits should be analyzed. It is important to highlight that no great weight of inference can be assigned to the fact that the audiometric threshold was a significant predictor in some models where age and noise exposure were not; individual hearing thresholds were entered because they were highly correlated with the dependent variable. The aim of this analysis was to determine if, for some tasks, age and noise exposure were significant predictors of performance once audiometric thresholds had been controlled for, and whether there was any evidence that the initial DTT model was being largely driven by hearing and not, in fact, noise exposure or age. The analysis does indicate that assuming listeners with a possible 30-dB dynamic range in their hearing thresholds as a homogeneous group could lead to erroneous results.

### Future Directions for Synaptopathy Research in Humans

Over the past 5 years, the work in our laboratory has focussed on investigating the presence and consequences of noise-induced cochlear synaptopathy in audiometrically normal young adults. We have tested healthy individuals with no hearing problems (Prendergast et al., 2017a, 2017b, 2018), listeners who report significant listening difficulties (Guest et al., 2018b), and listeners who report perceiving prolonged spontaneous tinnitus (Guest et al., 2017). In all instances, the initial work in the mouse model of cochlear synaptopathy (the low-SR hypothesis) was our starting point and formed the core of our assumptions regarding its likely manifestation in humans. In none of our studies do we find any strong evidence that electrophysiological measures of the auditory system vary systematically with noise exposure, nor do we find any perceptual consequences of increased levels of noise exposure. These findings are mirrored by the findings of the field more generally; even when there is evidence for the presence of noise-induced cochlear synaptopathy, the perceptual consequences of this are not apparent (Bramhall et al., 2019; Le Prell, 2019).

Subjective recall of a participant’s lifetime noise exposure is suboptimal as the main predictive metric in an analysis. However, after building up extensive experience in administering the Noise Exposure Structured Interview in our laboratory, we believe this is unlikely to be the main reason for our conclusions consistently showing a lack of relation between noise exposure and altered auditory performance. Guest et al. (2017) demonstrated that there is a statistically significant difference in Noise Exposure Structured Interview score for those with tinnitus compared with those without, and Ferguson, Tomlinson, Davis, and Lutman (2019) validated the lack of bias when using retrospective recall of speech communication effort to estimate levels of noise encountered in the real world. More likely is that participants who expose themselves to sufficient noise levels to result in a substantial noise-induced loss of cochlear synapses also sustain an audiometric threshold shift and thus are excluded from our research cohorts.

What may be required is a thorough consideration of the different assumptions underpinning our expectation of how cochlear synaptopathy might manifest itself in humans, and therefore the methodological techniques that can best elucidate this physiological change. The exploratory analyses presented here suggest that using Wave I as a proxy measure for the degree of synaptopathy does not result in clearer relations than those observed using estimated lifetime noise exposure as a proxy for the degree of synaptopathy. Furthermore, none of the measures vary as a function of age as predicted, which highlights the potential lack of sensitivity that exists for these measures. The between-subject variability of young listeners is high compared with the variance across the life span. It may be necessary to better understand the different sources of variability in the responses, and only if the variability across participants in the same parts of the age and noise exposure continuums (i.e., within low- and high-noise exposure groups and young and old listeners) can be reduced, will it be possible to reliably examine differences across different ages and levels of noise exposure. Unless this cross-participant variability within the same age range can be reduced, it will be difficult to study either noise-induced or age-related cochlear synaptopathy noninvasively using these techniques. It may be that the DTT and AMD measures could be used in combination to provide an insight into the health of the neural pathway to complement the snapshot of auditory health obtained via an audiogram. But currently, it is difficult to know if these responses are able to tell us about synaptopathy and, if so, the extent to which this is noise-induced or age-related.

It is also the case that for some of our tasks, such as AMD and the EFR, we do not adequately control or consider the role of the spread of excitation and the different coding mechanisms which could be at work. The analyses presented in this article demonstrate how *normal* hearing thresholds have a significant effect on dependent variables, and such relations are often overlooked or not accounted for in the literature on noise-induced cochlear synaptopathy in humans.

Human temporal bone studies are difficult to coordinate and time-consuming to perform, but through these, we are slowly gaining a better understanding of the rate of synaptic loss across the life span. The evidence that humans lose neural connections as a function of age is much clearer than the evidence that we lose them due to noise exposure and so a challenge is to now focus on older listeners who do not have normal audiometric function. There are potentially significant gains to be made in health care by developing tests of auditory function that can complement the audiogram to provide a complete picture of how different changes in the auditory system and different fiber groups can be assessed, leading to a more individualized process of diagnosing the state of a listener’s auditory health. However, it is our view that the assumptions that have underpinned the early work on cochlear synaptopathy in humans, including the measures used, must be challenged.
